# Supplementary material for: Using simulation model as a tool for analyzing bus service reliability and implementing improvement strategies
Source: PLoS One. 2020 May 7;15(5):e0232799. doi: 10.1371/journal.pone.0232799 (PMC7205290; doi:10.1371/journal.pone.0232799)
Supplement: S1 Dataset — (ZIP) [file pone.0232799.s001.zip › APPENDIX A-Codes.docx]

**APPENDIX A**

**SIMULATION MODEL CODE LISTING**

rm(list = ls())

#-------------------------------------- Source --------------------------------

source("RPKL Functions.R")

#-------------------------------------- Data ----------------------------------

load("RPKL Simulation running time data.RData")

load("RPKL Simulation dwell time data.RData")

load("RPKL Simulation headway data.RData")

load("RPKL Simulation terminal departure data.RData")

load("RPKL Simulation passenger demand data.RData")

t0 <- Sys.time()

#-------------------------------------- Input ---------------------------------

set.seed(0)

iteration <- 1000

nbus <- 9

### Sensitivity

SensitivityPassengerDemand <- 1

SensitivityTerminalDepartureDeviation <- 1

SensitivityDwellTimeVariation <- 1

SensitivityHeadwayVariation <- 1

PassengerArrivalRate <- 2

### Strategy

StrategyTerminalSchedule <- 0

StrategyTerminalHeadway <- 0

StrategyKeystopOneBus <- 0

StrategyKeystopTwoBus <- 0

### Preliminary

TerminalDepartureSchedule <- c(ctt("6:10:00"),

ctt("6:20:00"),

ctt("6:30:00"),

ctt("6:40:00"),

ctt("6:50:00"),

ctt("7:00:00"),

ctt("7:15:00"),

ctt("7:30:00"),

ctt("7:45:00"),

ctt("8:00:00"),

ctt("8:15:00"),

ctt("8:30:00"),

ctt("8:45:00"),

ctt("9:00:00"))

RunningTimeSchedule <- data.frame(matrix(c(600, 600, 600, 600, 600, 600,

600, 600, 600, 600, 600, 600,

600, 600, 600, 600, 600, 600,

600, 600, 600, 600, 600, 600,

600, 600, 600, 600, 600, 600,

600, 600, 600, 600, 600, 600,

600, 600, 600, 600, 600, 600,

600, 600, 600, 600, 600, 600,

600, 600, 600, 600, 600, 600), nrow = nbus))

HeadwaySchedule <- data.frame(matrix(c(600, 600, 600, 600, 600,

600, 600, 600, 600, 600,

600, 600, 600, 600, 600,

600, 600, 600, 600, 600,

600, 600, 600, 600, 600,

600, 600, 600, 600, 600,

600, 600, 600, 600, 600,

600, 600, 600, 600, 600), nrow = (nbus - 1)))

WaitingTimeSchedule <- 300

#-------------------------------------- Algorithm -----------------------------

MeanDwellTimeS <- c()

SDDwellTimeS <- c()

MeanDwellTimeS1 <- c()

MeanDwellTimeS2 <- c()

MeanDwellTimeS3 <- c()

SDDwellTimeS1 <- c()

SDDwellTimeS2 <- c()

SDDwellTimeS3 <- c()

MeanRunningTimeS1 <- c()

MeanRunningTimeS2 <- c()

MeanRunningTimeS3 <- c()

SDRunningTimeS1 <- c()

SDRunningTimeS2 <- c()

SDRunningTimeS3 <- c()

MeanHeadwayS <- c()

SDHeadwayS <- c()

MeanHeadwayS1 <- c()

MeanHeadwayS2 <- c()

MeanHeadwayS3 <- c()

SDHeadwayS1 <- c()

SDHeadwayS2 <- c()

SDHeadwayS3 <- c()

RejectDwellTime <- c()

RejectRunningTime <- c()

RejectHeadway <- c()

Output <- data.frame()

for(r in 1:iteration) {

#-------------------------------------- Tables --------------------------------

### Dwell time

attach(dwelltimedata)

fitks1 <- lm(Dwelltime[Keystop == 1] ~ Onboard[Keystop == 1] +

Boarding[Keystop == 1] +

Alighting[Keystop == 1])$coefficients

fitks2 <- lm(Dwelltime[Keystop == 2] ~ Onboard[Keystop == 2] +

Boarding[Keystop == 2] +

Alighting[Keystop == 2])$coefficients

fitks3 <- lm(Dwelltime[Keystop == 3] ~ Onboard[Keystop == 3] +

Boarding[Keystop == 3] +

Alighting[Keystop == 3])$coefficients

Passengerks1O <- cbind(1,

sample(Onboard[Keystop == 1], nbus, replace = T),

sample(Boarding[Keystop == 1], nbus, replace = T),

sample(Alighting[Keystop == 1], nbus, replace = T))

Passengerks1I <- cbind(1,

sample(Onboard[Keystop == 1], nbus, replace = T),

sample(Boarding[Keystop == 1], nbus, replace = T),

sample(Alighting[Keystop == 1], nbus, replace = T))

Passengerks2O <- cbind(1,

sample(Onboard[Keystop == 2], nbus, replace = T),

sample(Boarding[Keystop == 2], nbus, replace = T),

sample(Alighting[Keystop == 2], nbus, replace = T))

Passengerks2I <- cbind(1,

sample(Onboard[Keystop == 2], nbus, replace = T),

sample(Boarding[Keystop == 2], nbus, replace = T),

sample(Alighting[Keystop == 2], nbus, replace = T))

Passengerks3O <- cbind(1,

sample(Onboard[Keystop == 3], nbus, replace = T),

sample(Boarding[Keystop == 3], nbus, replace = T),

sample(Alighting[Keystop == 3], nbus, replace = T))

Boardings <- data.frame(KS1O = Passengerks1O[, 3],

KS2O = Passengerks2O[, 3],

KS3O = Passengerks3O[, 3],

KS2I = Passengerks2I[, 3],

KS1I = Passengerks1I[, 3])

Dwelltime1 <- Dwelltime[Keystop == 1]

Dwelltime2 <- Dwelltime[Keystop == 2]

Dwelltime3 <- Dwelltime[Keystop == 3]

detach(dwelltimedata)

DwellTimeKeystop <- round(cbind(Passengerks1O %*% fitks1,

Passengerks2O %*% fitks2,

Passengerks3O %*% fitks3,

Passengerks2I %*% fitks2,

Passengerks1I %*% fitks1))

colnames(DwellTimeKeystop) <- c("KS1O", "KS2O", "KS3O", "KS2I", "KS1I")

rownames(DwellTimeKeystop) <- sapply(1:nbus, function(x) paste("BUS", x, sep = ""))

attach(passengerdemanddata)

Passengerseg1O <- cbind(1,

Passengerks1O[, 2],

sample(b[d == 1 & s == 1], nbus, replace = T),

sample(a[d == 1 & s == 1], nbus, replace = T))

Passengerseg1I <- cbind(1,

Passengerks1I[, 2],

sample(b[d == 2 & s == 1], nbus, replace = T),

sample(a[d == 2 & s == 1], nbus, replace = T))

Passengerseg2O <- cbind(1,

Passengerks2O[, 2],

sample(b[d == 1 & s == 2], nbus, replace = T),

sample(a[d == 1 & s == 2], nbus, replace = T))

Passengerseg2I <- cbind(1,

Passengerks2I[, 2],

sample(b[d == 2 & s == 2], nbus, replace = T),

sample(a[d == 2 & s == 2], nbus, replace = T))

Passengerseg3O <- cbind(1,

Passengerks3O[, 2],

sample(b[d == 1 & s == 3], nbus, replace = T),

sample(a[d == 1 & s == 3], nbus, replace = T))

Passengerseg3I <- cbind(1,

Passengerks3O[, 2],

sample(b[d == 2 & s == 3], nbus, replace = T),

sample(a[d == 2 & s == 3], nbus, replace = T))

detach(passengerdemanddata)

DwellTimeSegment <- round(cbind(Passengerseg1O %*% fitks1,

Passengerseg2O %*% fitks2,

Passengerseg3O %*% fitks3,

Passengerseg3I %*% fitks3,

Passengerseg2I %*% fitks2,

Passengerseg1I %*% fitks1))

colnames(DwellTimeSegment) <- c("SEG1O", "SEG2O", "SEG3O", "SEG3I", "SEG2I", "SEG1I")

rownames(DwellTimeSegment) <- sapply(1:nbus, function(x) paste("BUS", x, sep = ""))

### Crowding

CrowdingSegment <- round(cbind(Passengerseg1O[, 2],

Passengerseg2O[, 2],

Passengerseg3O[, 2],

Passengerseg3I[, 2],

Passengerseg2I[, 2],

Passengerseg1I[, 2]))

colnames(CrowdingSegment) <- c("SEG1O", "SEG2O", "SEG3O", "SEG3I", "SEG2I", "SEG1I")

MeanCrowdingSegment <- round(mean(CrowdingSegment))

CrowdingKeystop <- round(cbind(Passengerks1O[, 2] + Passengerks1O[, 3] - Passengerks1O[, 4],

Passengerks2O[, 2] + Passengerks2O[, 3] - Passengerks2O[, 4],

Passengerks3O[, 2] + Passengerks3O[, 3] - Passengerks3O[, 4],

Passengerks2I[, 2] + Passengerks2I[, 3] - Passengerks2I[, 4],

Passengerks1I[, 2] + Passengerks1I[, 3] - Passengerks1I[, 4]))

colnames(CrowdingKeystop) <- c("KS1O", "KS2O", "KS3O", "KS2I", "KS1I")

MeanCrowdingKeystop <- round(mean(CrowdingKeystop))

### Running time

RunningTimeSegment <- data.frame(SEG1O = sample(runningtimedata[runningtimedata[, 2] == 1 & runningtimedata[, 1] <= 2400, 1], nbus, replace = T),

SEG2O = sample(runningtimedata[runningtimedata[, 2] == 2 & runningtimedata[, 1] <= 1200, 1], nbus, replace = T),

SEG3O = sample(runningtimedata[runningtimedata[, 2] == 3 & runningtimedata[, 1] <= 1800, 1], nbus, replace = T),

SEG3I = sample(runningtimedata[runningtimedata[, 2] == 3 & runningtimedata[, 1] <= 1800, 1], nbus, replace = T),

SEG2I = sample(runningtimedata[runningtimedata[, 2] == 2 & runningtimedata[, 1] <= 1200, 1], nbus, replace = T),

SEG1I = sample(runningtimedata[runningtimedata[, 2] == 1 & runningtimedata[, 1] <= 2400, 1] , nbus, replace = T))

rownames(RunningTimeSegment) <- sapply(1:nbus, function(x) paste("BUS", x, sep = ""))

colnames(RunningTimeSchedule) <- colnames(RunningTimeSegment)

rownames(RunningTimeSchedule) <- rownames(RunningTimeSegment)

Runningtime1 <- runningtimedata[runningtimedata[, 2] == 1, 1]

Runningtime2 <- runningtimedata[runningtimedata[, 2] == 2, 1]

Runningtime3 <- runningtimedata[runningtimedata[, 2] == 3, 1]

### Headway

if(StrategyTerminalSchedule == 1) {

TerminalDeparture <- TerminalDepartureSchedule[1:nbus]

} else {

TerminalDeparture <- sapply(sapply(1:nbus, function(x) sample(terminaldeparturedata[, x], 1)), function(y) ctt(y))

}

if(StrategyTerminalHeadway == 1) {

for(i0 in 1:(nbus - 1)) {

TerminalDeparture[i0 + 1] <- TerminalDeparture[i0] + HeadwaySchedule[i0, 1]

}

}

names(TerminalDeparture) <- NULL

ArrivalTimeKeystop <- cbind(TerminalDeparture, RunningTimeSegment)

colnames(ArrivalTimeKeystop) <- c("TEO", "KS1O", "KS2O", "KS3O", "KS2I", "KS1I", "TEI")

ArrivalTimeKeystop <- t(apply(ArrivalTimeKeystop, 1, cumsum))

if(StrategyKeystopOneBus == 1) {

alphaH <- 0.8 * 600

DepartureTimeKeystop <- ArrivalTimeKeystop[, 2:6] + DwellTimeKeystop

DepartureTimeKeystop <- DepartureTimeKeystop[-1, ]

ExpectedArrivalTimeKeystop <- ArrivalTimeKeystop[, 2:6] + alphaH

ExpectedArrivalTimeKeystop <- ExpectedArrivalTimeKeystop[-nbus, ]

rownames(ExpectedArrivalTimeKeystop) <- rownames(ArrivalTimeKeystop)[-1]

for(j1 in 1:5) {

for(j2 in 1:(nbus - 1)) {

if(ExpectedArrivalTimeKeystop[j2, j1] > DepartureTimeKeystop[j2, j1]) {

Holding <- min(180, ExpectedArrivalTimeKeystop[j2, j1] - DepartureTimeKeystop[j2, j1])

DepartureTimeKeystop[j2, j1:5] <- DepartureTimeKeystop[j2, j1:5] + Holding

ExpectedArrivalTimeKeystop[j2, j1:5] <- ExpectedArrivalTimeKeystop[j2, j1:5] + Holding

ArrivalTimeKeystop[j2 + 1, (j1 + 2):7] <- ArrivalTimeKeystop[j2 + 1, (j1 + 2):7] + Holding

}

}

}

}

if(StrategyKeystopTwoBus == 1) {

alphaH <- 0.8 * 600

DepartureTimeKeystop <- ArrivalTimeKeystop[, 2:6] + DwellTimeKeystop

DepartureTimeKeystop <- DepartureTimeKeystop[-1, ]

ExpectedArrivalTimeKeystop1 <- ArrivalTimeKeystop[, 2:6] + alphaH

ExpectedArrivalTimeKeystop1 <- ExpectedArrivalTimeKeystop1[-nbus, ]

rownames(ExpectedArrivalTimeKeystop1) <- rownames(ArrivalTimeKeystop)[-1]

ExpectedArrivalTimeKeystop2 <- ArrivalTimeKeystop[, 2:6] + RunningTimeSchedule[, 1:5]

ExpectedArrivalTimeKeystop2 <- ExpectedArrivalTimeKeystop2[-c(1,2), -5]

ExpectedArrivalTimeKeystop2 <- cbind(0, ExpectedArrivalTimeKeystop2)

colnames(ExpectedArrivalTimeKeystop2) <- colnames(ExpectedArrivalTimeKeystop1)

ExpectedArrivalTimeKeystop2[, -1] <- round((ExpectedArrivalTimeKeystop2[, -1] + ArrivalTimeKeystop[-c(nbus - 1, nbus), 3:6]) / 2)

ExpectedArrivalTimeKeystop2[, 1] <- ExpectedArrivalTimeKeystop1[1:(nbus - 2), 1]

ExpectedArrivalTimeKeystop2 <- rbind(ExpectedArrivalTimeKeystop2, ExpectedArrivalTimeKeystop1[nbus - 1, ])

rownames(ExpectedArrivalTimeKeystop2) <- rownames(ExpectedArrivalTimeKeystop1)

for(j1 in 1:5) {

for(j2 in 1:(nbus - 1)) {

ExpectedArrivalTimeKeystop <- min(ExpectedArrivalTimeKeystop1[j2, j1], ExpectedArrivalTimeKeystop2[j2, j1])

if(ExpectedArrivalTimeKeystop > DepartureTimeKeystop[j2, j1]) {

Holding <- min(180, ExpectedArrivalTimeKeystop - DepartureTimeKeystop[j2, j1])

DepartureTimeKeystop[j2, j1:5] <- DepartureTimeKeystop[j2, j1:5] + Holding

ExpectedArrivalTimeKeystop1[j2, j1:5] <- ExpectedArrivalTimeKeystop1[j2, j1:5] + Holding

ArrivalTimeKeystop[j2 + 1, (j1 + 2):7] <- ArrivalTimeKeystop[j2 + 1, (j1 + 2):7] + Holding

ExpectedArrivalTimeKeystop2 <- ArrivalTimeKeystop[, 2:6] + RunningTimeSchedule[, 1:5]

ExpectedArrivalTimeKeystop2 <- ExpectedArrivalTimeKeystop2[-c(1,2), -5]

ExpectedArrivalTimeKeystop2 <- cbind(0, ExpectedArrivalTimeKeystop2)

colnames(ExpectedArrivalTimeKeystop2) <- colnames(ExpectedArrivalTimeKeystop1)

ExpectedArrivalTimeKeystop2[, -1] <- round((ExpectedArrivalTimeKeystop2[, -1] + ArrivalTimeKeystop[-c(nbus - 1, nbus), 3:6]) / 2)

ExpectedArrivalTimeKeystop2[, 1] <- ExpectedArrivalTimeKeystop1[1:(nbus - 2), 1]

ExpectedArrivalTimeKeystop2 <- rbind(ExpectedArrivalTimeKeystop2, ExpectedArrivalTimeKeystop1[nbus - 1, ])

rownames(ExpectedArrivalTimeKeystop2) <- rownames(ExpectedArrivalTimeKeystop1)

}

}

}

}

ArrivalTimeschedule <- cbind(TerminalDepartureSchedule[1:nbus], RunningTimeSchedule)

colnames(ArrivalTimeschedule) <- c("TEO", "KS1O", "KS2O", "KS3O", "KS2I", "KS1I", "TEI")

ArrivalTimeschedule <- t(apply(ArrivalTimeschedule, 1, cumsum))

OnTimePerformance <- ArrivalTimeKeystop - ArrivalTimeschedule

HeadwayKeystop <- ArrivalTimeKeystop

for(i1 in 2:nrow(HeadwayKeystop)) {

HeadwayKeystop[i1, ] <- ArrivalTimeKeystop[i1, ] - ArrivalTimeKeystop[i1 - 1, ]

}

HeadwayKeystop <- abs(HeadwayKeystop[-1, 2:6])

rownames(HeadwayKeystop) <- NULL

Headway1 <- headwaydata[headwaydata[, 2] == 1, 1]

Headway2 <- headwaydata[headwaydata[, 2] == 2, 1]

Headway3 <- headwaydata[headwaydata[, 2] == 3, 1]

### Waiting time

WaitingTimeKeystop <- round(apply(HeadwayKeystop ^ 2, 2, sum) / (2 * apply(HeadwayKeystop, 2, sum)))

ExcessWaitingTimeKeystop <- WaitingTimeKeystop - WaitingTimeSchedule

MeanWaitingTimeKeystop <- round(mean(WaitingTimeKeystop))

MeanExcessWaitingTimeKeystop <- round(mean(ExcessWaitingTimeKeystop))

### HRIS and HRIR

HeadwayTerminal <- c()

HeadwayTerminalSchedule <- c()

for(i2 in 1:(nbus - 1)) {

HeadwayTerminal[i2] <- TerminalDeparture[i2 + 1] - TerminalDeparture[i2]

HeadwayTerminalSchedule[i2] <- TerminalDepartureSchedule[i2 + 1] - TerminalDepartureSchedule[i2]

}

HRIR <- round(sum(abs(1 - HeadwayTerminal / HeadwayTerminalSchedule)) / length(HeadwayTerminalSchedule), 2)

HRIS <- sapply(1:5, function(x) round(sum(abs(1 - HeadwayKeystop[, x] / HeadwaySchedule[, x])) / length(HeadwayKeystop[, x]), 2))

### Bunching and Big-gap

Bunching <- matrix(sapply(unlist(HeadwayKeystop), function(x) ifelse(x < 60, 1, 0)), nrow = nrow(HeadwayKeystop))

colnames(Bunching) <- colnames(HeadwayKeystop)

SumBunching <- sum(Bunching)

BigGap <- matrix(sapply(unlist(HeadwayKeystop), function(x) ifelse(x > 1800, 1, 0)), nrow = nrow(HeadwayKeystop))

colnames(BigGap) <- colnames(HeadwayKeystop)

SumBigGap <- sum(BigGap)

#-------------------------------------- Sensitivity ---------------------------

### Sensitivity Dwell time

SensitivityPassengerseg1O <- cbind(1, Passengerseg1O[, 2:4] * SensitivityPassengerDemand)

SensitivityPassengerseg1I <- cbind(1, Passengerseg1I[, 2:4] * SensitivityPassengerDemand)

SensitivityPassengerseg2O <- cbind(1, Passengerseg2O[, 2:4] * SensitivityPassengerDemand)

SensitivityPassengerseg2I <- cbind(1, Passengerseg2I[, 2:4] * SensitivityPassengerDemand)

SensitivityPassengerseg3O <- cbind(1, Passengerseg3O[, 2:4] * SensitivityPassengerDemand)

SensitivityPassengerseg3I <- cbind(1, Passengerseg3I[, 2:4] * SensitivityPassengerDemand)

SensitivityPassengerks1O <- cbind(1, Passengerks1O[, 2:4] * SensitivityPassengerDemand)

SensitivityPassengerks1I <- cbind(1, Passengerks1I[, 2:4] * SensitivityPassengerDemand)

SensitivityPassengerks2O <- cbind(1, Passengerks2O[, 2:4] * SensitivityPassengerDemand)

SensitivityPassengerks2I <- cbind(1, Passengerks2I[, 2:4] * SensitivityPassengerDemand)

SensitivityPassengerks3O <- cbind(1, Passengerks3O[, 2:4] * SensitivityPassengerDemand)

SensitivityDwellTimeks0 <- round(cbind(SensitivityPassengerks1O %*% fitks1,

SensitivityPassengerks2O %*% fitks2,

SensitivityPassengerks3O %*% fitks3,

SensitivityPassengerks2I %*% fitks2,

SensitivityPassengerks1I %*% fitks1))

colnames(SensitivityDwellTimeks0) <- c("KS1O", "KS2O", "KS3O", "KS2I", "KS1I")

rownames(SensitivityDwellTimeks0) <- sapply(1:nbus, function(x) paste("BUS", x, sep = ""))

SensitivityDwellTimeseg0 <- round(cbind(SensitivityPassengerseg1O %*% fitks1,

SensitivityPassengerseg2O %*% fitks2,

SensitivityPassengerseg3O %*% fitks3,

SensitivityPassengerseg3I %*% fitks3,

SensitivityPassengerseg2I %*% fitks2,

SensitivityPassengerseg1I %*% fitks1))

colnames(SensitivityDwellTimeseg0) <- c("SEG1O", "SEG2O", "SEG3O", "SEG3I", "SEG2I", "SEG1I")

rownames(SensitivityDwellTimeseg0) <- sapply(1:nbus, function(x) paste("BUS", x, sep = ""))

DwellTimeDifferenceks0 <- SensitivityDwellTimeks0 - DwellTimeKeystop

DwellTimeDifferenceseg0 <- SensitivityDwellTimeseg0 - DwellTimeSegment

SensitivityPassengerksArrival1 <- (t(apply(DwellTimeDifferenceseg0, 1, cumsum)) %/% 60) * PassengerArrivalRate

temp <- c()

for(i3 in 1:5) {

temp <- cbind(temp, DwellTimeDifferenceseg0[, i3], DwellTimeDifferenceks0[, i3])

}

SensitivityPassengerArrival <- (t(apply(temp, 1, cumsum)) %/% 60) * PassengerArrivalRate

SensitivityDwellTime <- round(cbind((SensitivityPassengerseg1O) %*% fitks1,

(SensitivityPassengerks1O + cbind(0, 0, SensitivityPassengerArrival[, 1], 0)) %*% fitks1,

(SensitivityPassengerseg2O + cbind(0, 0, SensitivityPassengerArrival[, 2], 0)) %*% fitks2,

(SensitivityPassengerks2O + cbind(0, 0, SensitivityPassengerArrival[, 3], 0)) %*% fitks2,

(SensitivityPassengerseg3O + cbind(0, 0, SensitivityPassengerArrival[, 4], 0)) %*% fitks3,

(SensitivityPassengerks3O + cbind(0, 0, SensitivityPassengerArrival[, 5], 0)) %*% fitks3,

(SensitivityPassengerseg3I + cbind(0, 0, SensitivityPassengerArrival[, 6], 0)) %*% fitks3,

(SensitivityPassengerks2I + cbind(0, 0, SensitivityPassengerArrival[, 7], 0)) %*% fitks2,

(SensitivityPassengerseg2I + cbind(0, 0, SensitivityPassengerArrival[, 8], 0)) %*% fitks2,

(SensitivityPassengerks1I + cbind(0, 0, SensitivityPassengerArrival[, 9], 0)) %*% fitks1,

(SensitivityPassengerseg1I + cbind(0, 0, SensitivityPassengerArrival[, 10], 0)) %*% fitks1))

SensitivityDwellTime <- SensitivityDwellTimeVariation * SensitivityDwellTime

colnames(SensitivityDwellTime) <- c("SEG1O", "KS1O", "SEG2O", "KS2O", "SEG3O", "KS3O", "SEG3I", "KS2I", "SEG2I", "KS1I", "SEG1I")

rownames(SensitivityDwellTime) <- sapply(1:nbus, function(x) paste("BUS", x, sep = ""))

DwellTimeDifferenceKeystop <- SensitivityDwellTime[, c(2, 4, 6, 8, 10)] - DwellTimeKeystop

DwellTimeDifferenceSegment <- SensitivityDwellTime[, c(1, 3, 5, 7,9, 11)] - DwellTimeSegment

### Sensitivity Crowding

SensitivityCrowdingSegment <- round(cbind(SensitivityPassengerseg1O[, 2],

SensitivityPassengerseg2O[, 2],

SensitivityPassengerseg3O[, 2],

SensitivityPassengerseg3I[, 2],

SensitivityPassengerseg2I[, 2],

SensitivityPassengerseg1I[, 2]))

colnames(SensitivityCrowdingSegment) <- c("SEG1O", "SEG2O", "SEG3O", "SEG3I", "SEG2I", "SEG1I")

MeanSensitivityCrowdingSegment <- round(mean(SensitivityCrowdingSegment))

SensitivityCrowdingKeystop <- abs(round(cbind(SensitivityPassengerks1O[, 2] + SensitivityPassengerks1O[, 3] - SensitivityPassengerks1O[, 4],

SensitivityPassengerks2O[, 2] + SensitivityPassengerks2O[, 3] - SensitivityPassengerks2O[, 4],

SensitivityPassengerks3O[, 2] + SensitivityPassengerks3O[, 3] - SensitivityPassengerks3O[, 4],

SensitivityPassengerks2I[, 2] + SensitivityPassengerks2I[, 3] - SensitivityPassengerks2I[, 4],

SensitivityPassengerks1I[, 2] + SensitivityPassengerks1I[, 3] - SensitivityPassengerks1I[, 4])))

colnames(SensitivityCrowdingKeystop) <- c("KS1O", "KS2O", "KS3O", "KS2I", "KS1I")

MeanSensitivityCrowdingKeystop <- round(mean(SensitivityCrowdingKeystop))

### Sensitivity Running time

SensitivityRunningTimeSegment <- RunningTimeSegment + DwellTimeDifferenceSegment + cbind(0, DwellTimeDifferenceKeystop)

### Sensitivity Headway

TerminalDepartureDifference <- TerminalDeparture - TerminalDepartureSchedule[1:nbus]

SensitivityTerminalDeparture <- TerminalDepartureSchedule[1:nbus] + round(SensitivityTerminalDepartureDeviation * TerminalDepartureDifference)

SensitivityArrivalTimeKeystop <- cbind(SensitivityTerminalDeparture, SensitivityRunningTimeSegment)

colnames(SensitivityArrivalTimeKeystop) <- c("TEO", "KS1O", "KS2O", "KS3O", "KS2I", "KS1I", "TEI")

SensitivityArrivalTimeKeystop <- t(apply(SensitivityArrivalTimeKeystop, 1, cumsum))

SensitivityOnTimePerformance <- SensitivityArrivalTimeKeystop - ArrivalTimeschedule

SensitivityHeadwayKeystop <- SensitivityArrivalTimeKeystop

for(i4 in 2:nrow(SensitivityHeadwayKeystop)) {

SensitivityHeadwayKeystop[i4, ] <- SensitivityArrivalTimeKeystop[i4, ] - SensitivityArrivalTimeKeystop[i4 - 1, ]

}

SensitivityHeadwayKeystop <- abs(SensitivityHeadwayKeystop[-1, 2:6])

SensitivityHeadwayKeystop <- SensitivityHeadwayVariation * SensitivityHeadwayKeystop

rownames(SensitivityHeadwayKeystop) <- NULL

### Sensitivity Waiting time

SensitivityWaitingTimeKeystop <- round(apply(SensitivityHeadwayKeystop ^ 2, 2, sum) / (2 * apply(SensitivityHeadwayKeystop, 2, sum)))

SensitivityExcessWaitingTimeKeystop <- SensitivityWaitingTimeKeystop - WaitingTimeSchedule

MeanSensitivityWaitingTimeKeystop <- round(mean(SensitivityWaitingTimeKeystop))

MeanSensitivityExcessWaitingTimeKeystop <- round(mean(SensitivityExcessWaitingTimeKeystop))

### HRIS and HRIR

SensitivityHeadwayTerminal <- c()

SensitivityHeadwayTerminalSchedule <- c()

for(i5 in 1:(nbus - 1)) {

SensitivityHeadwayTerminal[i5] <- SensitivityTerminalDeparture[i5 + 1] - SensitivityTerminalDeparture[i5]

SensitivityHeadwayTerminalSchedule[i5] <- TerminalDepartureSchedule[i5 + 1] - TerminalDepartureSchedule[i5]

}

SensitivityHRIR <- round(sum(abs(1 - SensitivityHeadwayTerminal / SensitivityHeadwayTerminalSchedule)) / length(SensitivityHeadwayTerminalSchedule), 2)

SensitivityHRIS <- sapply(1:5, function(x) round(sum(abs(1 - SensitivityHeadwayKeystop[, x] / HeadwaySchedule[, x])) / length(SensitivityHeadwayKeystop[, x]), 2))

### Sensitivity Bunching and Big-gap

SensitivityBunching <- matrix(sapply(unlist(SensitivityHeadwayKeystop), function(x) ifelse(x < 60, 1, 0)), nrow = nrow(SensitivityHeadwayKeystop))

colnames(SensitivityBunching) <- colnames(SensitivityHeadwayKeystop)

SumSensitivityBunching <- sum(SensitivityBunching)

SensitivityBigGap <- matrix(sapply(unlist(SensitivityHeadwayKeystop), function(x) ifelse(x > 1800, 1, 0)), nrow = nrow(SensitivityHeadwayKeystop))

colnames(SensitivityBigGap) <- colnames(SensitivityHeadwayKeystop)

SumSensitivityBigGap <- sum(SensitivityBigGap)

#-------------------------------------- Validation ----------------------------

### Dwell time

Pvdw <- c()

Pvdw[1] <- wilcox.test(DwellTimeKeystop[, 1], Dwelltime1, exact = F)$p.value

Pvdw[2] <- wilcox.test(DwellTimeKeystop[, 2], Dwelltime2, exact = F)$p.value

Pvdw[3] <- wilcox.test(DwellTimeKeystop[, 3], Dwelltime3, exact = F)$p.value

Pvdw[4] <- wilcox.test(DwellTimeKeystop[, 4], Dwelltime2, exact = F)$p.value

Pvdw[5] <- wilcox.test(DwellTimeKeystop[, 5], Dwelltime1, exact = F)$p.value

RejectDwellTime <- rbind(RejectDwellTime, Pvdw)

MeanDwellTimeP1 <- round(mean(Dwelltime1))

MeanDwellTimeP2 <- round(mean(Dwelltime2))

MeanDwellTimeP3 <- round(mean(Dwelltime3))

SDDwellTimeP1 <- round(sd(Dwelltime1))

SDDwellTimeP2 <- round(sd(Dwelltime2))

SDDwellTimeP3 <- round(sd(Dwelltime3))

MeanDwellTimeS1[r] <- round(mean(unlist(DwellTimeKeystop[, c(1, 5)])))

MeanDwellTimeS2[r] <- round(mean(unlist(DwellTimeKeystop[, c(2, 4)])))

MeanDwellTimeS3[r] <- round(mean(unlist(DwellTimeKeystop[, 3])))

SDDwellTimeS1[r] <- round(sd(unlist(DwellTimeKeystop[, c(1, 5)])))

SDDwellTimeS2[r] <- round(sd(unlist(DwellTimeKeystop[, c(2, 4)])))

SDDwellTimeS3[r] <- round(sd(unlist(DwellTimeKeystop[, 3])))

### Running time

Pvru <- c()

Pvru[1] <- wilcox.test(RunningTimeSegment[, 1], Runningtime1, exact = F)$p.value

Pvru[2] <- wilcox.test(RunningTimeSegment[, 2], Runningtime2, exact = F)$p.value

Pvru[3] <- wilcox.test(RunningTimeSegment[, 3], Runningtime3, exact = F)$p.value

Pvru[4] <- wilcox.test(RunningTimeSegment[, 4], Runningtime3, exact = F)$p.value

Pvru[5] <- wilcox.test(RunningTimeSegment[, 5], Runningtime2, exact = F)$p.value

Pvru[6] <- wilcox.test(RunningTimeSegment[, 6], Runningtime1, exact = F)$p.value

RejectRunningTime <- rbind(RejectRunningTime, Pvru)

MeanRunningTimeP1 <- round(mean(Runningtime1))

MeanRunningTimeP2 <- round(mean(Runningtime2))

MeanRunningTimeP3 <- round(mean(Runningtime3))

SDRunningTimeP1 <- round(sd(Runningtime1))

SDRunningTimeP2 <- round(sd(Runningtime2))

SDRunningTimeP3 <- round(sd(Runningtime3))

MeanRunningTimeS1[r] <- round(mean(unlist(RunningTimeSegment[, c(1, 6)])))

MeanRunningTimeS2[r] <- round(mean(unlist(RunningTimeSegment[, c(2, 5)])))

MeanRunningTimeS3[r] <- round(mean(unlist(RunningTimeSegment[, c(3, 4)])))

SDRunningTimeS1[r] <- round(sd(unlist(RunningTimeSegment[, c(1, 6)])))

SDRunningTimeS2[r] <- round(sd(unlist(RunningTimeSegment[, c(2, 5)])))

SDRunningTimeS3[r] <- round(sd(unlist(RunningTimeSegment[, c(3, 4)])))

### Headway

Pvhw <- c()

Pvhw[1] <- wilcox.test(HeadwayKeystop[, 1], Headway1, exact = F)$p.value

Pvhw[2] <- wilcox.test(HeadwayKeystop[, 2], Headway2, exact = F)$p.value

Pvhw[3] <- wilcox.test(HeadwayKeystop[, 3], Headway3, exact = F)$p.value

Pvhw[4] <- wilcox.test(HeadwayKeystop[, 4], Headway2, exact = F)$p.value

Pvhw[5] <- wilcox.test(HeadwayKeystop[, 5], Headway1, exact = F)$p.value

RejectHeadway <- rbind(RejectHeadway, Pvhw)

MeanHeadwayP1 <- round(mean(Headway1))

MeanHeadwayP2 <- round(mean(Headway2))

MeanHeadwayP3 <- round(mean(Headway3))

SDHeadwayP1 <- round(sd(Headway1))

SDHeadwayP2 <- round(sd(Headway2))

SDHeadwayP3 <- round(sd(Headway3))

MeanHeadwayS1[r] <- round(mean(unlist(HeadwayKeystop[, c(1, 5)])))

MeanHeadwayS2[r] <- round(mean(unlist(HeadwayKeystop[, c(2, 4)])))

MeanHeadwayS3[r] <- round(mean(unlist(HeadwayKeystop[, 3])))

SDHeadwayS1[r] <- round(sd(unlist(HeadwayKeystop[, c(1, 5)])))

SDHeadwayS2[r] <- round(sd(unlist(HeadwayKeystop[, c(2, 4)])))

SDHeadwayS3[r] <- round(sd(unlist(HeadwayKeystop[, 3])))

#-------------------------------------- Results -------------------------------

### Reliability

Output[r, 1] <- MeanDwellTimeS1[r]

Output[r, 2] <- MeanDwellTimeP1

Output[r, 3] <- SDDwellTimeS1[r]

Output[r, 4] <- SDDwellTimeP1

Output[r, 5] <- MeanDwellTimeS2[r]

Output[r, 6] <- MeanDwellTimeP2

Output[r, 7] <- SDDwellTimeS2[r]

Output[r, 8] <- SDDwellTimeP2

Output[r, 9] <- MeanDwellTimeS3[r]

Output[r, 10] <- MeanDwellTimeP3

Output[r, 11] <- SDDwellTimeS3[r]

Output[r, 12] <- SDDwellTimeP3

Output[r, 13] <- MeanRunningTimeS1[r]

Output[r, 14] <- MeanRunningTimeP1

Output[r, 15] <- SDRunningTimeS1[r]

Output[r, 16] <- SDRunningTimeP1

Output[r, 17] <- MeanRunningTimeS2[r]

Output[r, 18] <- MeanRunningTimeP2

Output[r, 19] <- SDRunningTimeS2[r]

Output[r, 20] <- SDRunningTimeP2

Output[r, 21] <- MeanRunningTimeS3[r]

Output[r, 22] <- MeanRunningTimeP3

Output[r, 23] <- SDRunningTimeS3[r]

Output[r, 24] <- SDRunningTimeP3

Output[r, 25] <- MeanHeadwayS1[r]

Output[r, 26] <- MeanHeadwayP1

Output[r, 27] <- SDHeadwayS1[r]

Output[r, 28] <- SDHeadwayP1

Output[r, 29] <- MeanHeadwayS2[r]

Output[r, 30] <- MeanHeadwayP2

Output[r, 31] <- SDHeadwayS2[r]

Output[r, 32] <- SDHeadwayP2

Output[r, 33] <- MeanHeadwayS3[r]

Output[r, 34] <- MeanHeadwayP3

Output[r, 35] <- SDHeadwayS3[r]

Output[r, 36] <- SDHeadwayP3

Output[r, 37] <- ifelse(RejectDwellTime[r, 1] <= 0.05, 1, 0)

Output[r, 38] <- ifelse(RejectDwellTime[r, 2] <= 0.05, 1, 0)

Output[r, 39] <- ifelse(RejectDwellTime[r, 3] <= 0.05, 1, 0)

Output[r, 40] <- ifelse(RejectDwellTime[r, 4] <= 0.05, 1, 0)

Output[r, 41] <- ifelse(RejectDwellTime[r, 5] <= 0.05, 1, 0)

Output[r, 42] <- ifelse(RejectRunningTime[r, 1] <= 0.05, 1, 0)

Output[r, 43] <- ifelse(RejectRunningTime[r, 2] <= 0.05, 1, 0)

Output[r, 44] <- ifelse(RejectRunningTime[r, 3] <= 0.05, 1, 0)

Output[r, 45] <- ifelse(RejectRunningTime[r, 4] <= 0.05, 1, 0)

Output[r, 46] <- ifelse(RejectRunningTime[r, 5] <= 0.05, 1, 0)

Output[r, 47] <- ifelse(RejectRunningTime[r, 6] <= 0.05, 1, 0)

Output[r, 48] <- ifelse(RejectHeadway[r, 1] <= 0.05, 1, 0)

Output[r, 49] <- ifelse(RejectHeadway[r, 2] <= 0.05, 1, 0)

Output[r, 50] <- ifelse(RejectHeadway[r, 3] <= 0.05, 1, 0)

Output[r, 51] <- ifelse(RejectHeadway[r, 4] <= 0.05, 1, 0)

Output[r, 52] <- ifelse(RejectHeadway[r, 5] <= 0.05, 1, 0)

### Sensitivity

Output[r, 53] <- MeanSensitivityWaitingTimeKeystop

Output[r, 54] <- MeanSensitivityExcessWaitingTimeKeystop

Output[r, 55] <- MeanSensitivityCrowdingSegment

Output[r, 56] <- MeanSensitivityCrowdingKeystop

Output[r, 57] <- SensitivityHRIR

Output[r, 58] <- SensitivityHRIS[1]

Output[r, 59] <- SensitivityHRIS[2]

Output[r, 60] <- SensitivityHRIS[3]

Output[r, 61] <- SensitivityHRIS[4]

Output[r, 62] <- SensitivityHRIS[5]

Output[r, 63] <- SumSensitivityBigGap

Output[r, 64] <- SumSensitivityBunching

### Strategy

Output[r, 65] <- MeanWaitingTimeKeystop

Output[r, 66] <- MeanExcessWaitingTimeKeystop

Output[r, 67] <- MeanCrowdingSegment

Output[r, 68] <- MeanCrowdingKeystop

Output[r, 69] <- HRIR

Output[r, 70] <- HRIS[1]

Output[r, 71] <- HRIS[2]

Output[r, 72] <- HRIS[3]

Output[r, 73] <- HRIS[4]

Output[r, 74] <- HRIS[5]

Output[r, 75] <- SumBigGap

Output[r, 76] <- SumBunching

}

#-------------------------------------- Output --------------------------------

### Validation

# plot(Output[, 1], main = "Dwelltime")

# abline(h = Output[1, 2])

# plot(Output[, 5], main = "RunningTime")

# abline(h = Output[1, 6])

# plot(Output[, 5], main = "Headway")

# abline(h = Output[1, 6])

# hist(Output[, 1])

# hist(Output[, 3])

# hist(Output[, 5])

# boxplot(Output[, 1])

# boxplot(Output[, 3])

# boxplot(Output[, 5])

SummaryValidation <- data.frame("MeanDwellTimeS1" = round(mean(Output[, 1])),

"MeanDwellTimeP1" = round(mean(Output[, 2])),

"SDDwellTimeS1" = round(mean(Output[, 3])),

"SDDwellTimeP1" = round(mean(Output[, 4])),

"MeanDwellTimeS2" = round(mean(Output[, 5])),

"MeanDwellTimeP2" = round(mean(Output[, 6])),

"SDDwellTimeS2" = round(mean(Output[, 7])),

"SDDwellTimeP2" = round(mean(Output[, 8])),

"MeanDwellTimeS3" = round(mean(Output[, 9])),

"MeanDwellTimeP3" = round(mean(Output[, 10])),

"SDDwellTimeS3" = round(mean(Output[, 11])),

"SDDwellTimeP3" = round(mean(Output[, 12])),

"MeanRunningTimeS1" = round(mean(Output[, 13])),

"MeanRunningTimeP1" = round(mean(Output[, 14])),

"SDRunningTimeS1" = round(mean(Output[, 15])),

"SDRunningTimeP1" = round(mean(Output[, 16])),

"MeanRunningTimeS2" = round(mean(Output[, 17])),

"MeanRunningTimeP2" = round(mean(Output[, 18])),

"SDRunningTimeS2" = round(mean(Output[, 19])),

"SDRunningTimeP2" = round(mean(Output[, 20])),

"MeanRunningTimeS3" = round(mean(Output[, 21])),

"MeanRunningTimeP3" = round(mean(Output[, 22])),

"SDRunningTimeS3" = round(mean(Output[, 23])),

"SDRunningTimeP3" = round(mean(Output[, 24])),

"MeanHeadwayS1" = round(mean(Output[, 25])),

"MeanHeadwayP1" = round(mean(Output[, 26])),

"SDHeadwayS1" = round(mean(Output[, 27])),

"SDHeadwayP1" = round(mean(Output[, 28])),

"MeanHeadwayS2" = round(mean(Output[, 29])),

"MeanHeadwayP2" = round(mean(Output[, 30])),

"SDHeadwayS2" = round(mean(Output[, 31])),

"SDHeadwayP2" = round(mean(Output[, 32])),

"MeanHeadwayS3" = round(mean(Output[, 33])),

"MeanHeadwayP3" = round(mean(Output[, 34])),

"SDHeadwayS3" = round(mean(Output[, 35])),

"SDHeadwayP3" = round(mean(Output[, 36])),

"RejectDwellTime1" = sum(Output[, 37]),

"RejectDwellTime2" = sum(Output[, 38]),

"RejectDwellTime3" = sum(Output[, 39]),

"RejectDwellTime4" = sum(Output[, 40]),

"RejectDwellTime5" = sum(Output[, 41]),

"RejectRunningTime1" = sum(Output[, 42]),

"RejectRunningTime2" = sum(Output[, 43]),

"RejectRunningTime3" = sum(Output[, 44]),

"RejectRunningTime4" = sum(Output[, 45]),

"RejectRunningTime5" = sum(Output[, 46]),

"RejectRunningTime6" = sum(Output[, 47]),

"RejectHeadway1" = sum(Output[, 48]),

"RejectHeadway2" = sum(Output[, 49]),

"RejectHeadway3" = sum(Output[, 50]),

"RejectHeadway4" = sum(Output[, 51]),

"RejectHeadway5" = sum(Output[, 52]))

### Sensitivity

SensitivityCrowdingSegmentdata <- Output[, 55]

SensitivityCrowdingKeystopdata <- Output[, 56]

SummarySensitivity <- data.frame("MeanWaitingTime" = round(mean(Output[, 53])),

"MeanExcessWaitingTime" = round(mean(Output[, 54])),

"MeanCrowdingSegment" = round(mean(Output[, 55])),

"MeanCrowdingKeystop" = round(mean(Output[, 56])),

"MeanHRIR" = round(mean(Output[, 57]), 3),

"MeanHRIS1O" = round(mean(Output[, 58]), 3),

"MeanHRIS2O" = round(mean(Output[, 59]), 3),

"MeanHRIS3O" = round(mean(Output[, 60]), 3),

"MeanHRIS1I" = round(mean(Output[, 61]), 3),

"MeanHRIS2I" = round(mean(Output[, 62]), 3),

"MeanBigGap" = round(mean(Output[, 63])),

"MeanBunching" = round(mean(Output[, 64])))

### Strategy

StrategyCrowdingSegmentdata <- Output[, 67]

StrategyCrowdingKeystopdata <- Output[, 68]

SummaryStrategy <- data.frame("MeanWaitingTime" = round(mean(Output[, 65])),

"MeanExcessWaitingTime" = round(mean(Output[, 66])),

"MeanCrowdingSegment" = round(mean(Output[, 67])),

"MeanCrowdingKeystop" = round(mean(Output[, 68])),

"MeanHRIR" = round(mean(Output[, 69]), 3),

"MeanHRIS1O" = round(mean(Output[, 70]), 3),

"MeanHRIS2O" = round(mean(Output[, 71]), 3),

"MeanHRIS3O" = round(mean(Output[, 72]), 3),

"MeanHRIS1I" = round(mean(Output[, 73]), 3),

"MeanHRIS2I" = round(mean(Output[, 74]), 3),

"MeanBigGap" = round(mean(Output[, 75])),

"MeanBunching" = round(mean(Output[, 76])))

### Summary tables

summary(SensitivityCrowdingSegmentdata)

summary(SensitivityCrowdingKeystopdata)

summary(StrategyCrowdingSegmentdata)

summary(StrategyCrowdingKeystopdata)

SummaryValidation

SummarySensitivity

SummaryStrategy

Sys.time() - t0

# write.csv(SummaryValidation, file = "Summary Validation.CSV")

# write.csv(SummarySensitivity, file = "SummarySensitivityHeadVar-2.00.CSV")

# write.csv(CrowdingSegment, file = "CrowdingSegmentHeadVar-2.00.CSV")

# write.csv(CrowdingKeystop, file = "CrowdingKeystopHeadVar-2.00.CSV")
