# Supplementary material for: Using simulation model as a tool for analyzing bus service reliability and implementing improvement strategies
Source: PLoS One. 2020 May 7;15(5):e0232799. doi: 10.1371/journal.pone.0232799 (PMC7205290; doi:10.1371/journal.pone.0232799)
Supplement: S1 Dataset — (ZIP) [file pone.0232799.s001.zip › Datainbrief_template.docx]

**Article Title**

**Using simulation model as a tool for analysing bus service reliability and implementing improvement strategies**

**Authors**

**Seyed Mohammad Hossein Moosavi^*^(**[**mh.moosavi@um.edu.my**](mailto:mh.moosavi@um.edu.my)**)**

Centre for Transportation Research (CTR), Level 3, Block N, Faculty of Engineering, University of Malaya (UM), 50603 Kuala Lumpur, Malaysia.

**Amiruddin Ismail (**[**aismail@ukm.edu.my**](mailto:aismail@ukm.edu.my)**)**

Smart and Sustainable Township Reseach Centre, Faculty of Engineering, National university of Malaysia (UKM), 46300 Bandar Bangi, Malaysia

**Yuen Choon Wah (**[**yuencw@um.edu.my**](mailto:yuencw@um.edu.my)**)**

Centre for Transportation Research (CTR), Level 3, Block N, Faculty of Engineering, University of Malaya (UM)

**Peyman Babashamsi (**[**peymenshams@ukm.edu.my**](mailto:peymenshams@ukm.edu.my)**)**

Smart and Sustainable Township Reseach Centre, Faculty of Engineering, National university of Malaysia (UKM), 46300 Bandar Bangi, Malaysia

***Corresponding Author:**

**Seyed Mohammad Hossein Moosavi**

Centre for Transportation Research (CTR),

Level 3, Block N

Department of Civil Engineering,

Faculty of Engineering,

University of Malaya,

50603 Kuala Lumpur,

MALAYSIA.

Tel: +601137660889

e-mail 1: [mh.moosavi@um.edu.my](mailto:mh.moosavi@um.edu.my) e-mail2: [mh.moosavi65@gmail.com](mailto:mh.moosavi65@gmail.com)

**Abstract**

Service variation can greatly impact passengers’ experience and perception of bus services. In addition, unreliable service can increase operating costs and decrease revenue. According to review of literature, there is no consistency in reliability definition and indicators. Companies have their own definition of bus service reliability and they mostly neglect about the passengers’ perspective of reliability. Accordingly, four different reliability indicators were selected in this study to cover both passengers’ and operators’ perceptions of reliability: waiting time and on-board crowding level from passengers’ perspective, and headway regularity index (on-time performance) and bus bunching/big gap percentage from operators’ perspective. The primary objective of this research is to improve reliability of high-frequency bus service and analysis tools currently used in the bus transit industry. Therefore, a simulation model of high frequency bus service was developed in order to study the causes of service unreliability and strategies to alleviate it. Next, several operating strategies, including terminal departure and time-point holding for schedule or headway, were modelled and evaluated for their potential to improve reliability. The simulation model was verified with an interactive debugger, a detailed log file, and animated playback. The validation process compared real performance of a route to simulated performance with the same vehicle profile in terms of dwell times, segment running times, and headways. The primary data source for model calibration is the raw automatically collected data from Automatic Vehicle Location (AVL), Automatic Passenger Counting (APC) and Automatic Fare Collection (AFC) systems.

**Keywords**

Service Reliability, Simulation Model, improvement strategies, Headway Regularity, Waiting Time

**Specifications Table**

| **Subject** | Transportation |
| --- | --- |
| **Specific subject area** | Evaluating Bus Service Reliability Using Simulation Model |
| **Type of data** | Table  Image  Figure |
| **How data were acquired** | The primary data source for model calibration is the raw automatically collected data from Automatic Vehicle Location (AVL), Automatic Passenger Counting (APC) and Automatic Fare Collection (AFC) systems.  The simulation model itself is developed in RStudio. RStudio is a free and open-source integrated development environment (IDE) for R, a programming language for statistical computing and graphics. |
| **Data format** | Raw  Analyzed  Filtered |
| **Parameters for data collection** | Data collected for period of 3 months from RapidKL bus company archive. Only weekdays were considered. A high-frequency bus service route was selected which is located in Kuala Lampur City Centre. |
| **Description of data collection** | The primary data source for model calibration is the raw automatically collected data from Automatic Vehicle Location (AVL), Automatic Passenger Counting (APC) and Automatic Fare Collection (AFC) systems. The data from these systems is saved on the bus and transferred to the RapidKL database when the bus refuels.  Route U32 selected in order to conducting this study. This route passes across the most congested sections of the Kuala Lumpur City Centre (KLCC), providing a suitable environment for conducting study on service regularity and reliability. Route U32 is a high-frequency route with high passenger demand. This route has fifty-nine bus stops (almost 30 stops in each direction) and nine buses are operating in this route along an operation day. |
| **Data source location** | [Fill the available information in, and delete from this list as appropriate:  City/Town/Region: Kuala Lampure City Centre  Country: Malaysia Latitude and longitude (and GPS coordinates) for collected samples/data:] 3°08'39.5"N 101°42'20.1"E 3.144310, 101.705591 |
| **Data accessibility** | [State here if the data are either hosted ‘With the article’ or on a public repository. The journal does not have a strict policy but in the interest of openly sharing data we recommend hosting your data in a trusted repository. Please delete or complete as appropriate, either:]  With the article |

**Value of the Data**

- Why are these data useful?

Evolution of public road transportation systems requires analysis and planning tools to improve service quality. It is very difficult or even impossible to use direct experimentation considering legal, financial, material or time constraints in order to develop new public transportation solutions. Moreover, researchers cannot establish a theoretical model for such systems due to their size and complexity. Thus, computer simulation should be adapted as a solution for analysis and planning of public transportation systems.

Bus services naturally tend to be unstable and are unable to adhere to schedules without control strategies. Service providers and researchers need to know how implementing control strategies can improve service reliability. Researchers can gain clear insight by using analysed, modelled and simulated data provided in this research.

- Who can benefit from these data?

Urban public transportation researchers, experts and service providers.

- How can these data be used for further insights and development of experiments?

The practical framework is presented by this study to evaluate current level of service reliability, implement corrective strategies and evaluating the impact of strategies on service performance. This framework can be adapted to other routes and also to the rail transports (such as subways). Moreover, this there more strategies which can be developed and implement on routes, following the presented framework.

**1. Data**

The primary data source for model calibration is the raw automatically collected data from Automatic Vehicle Location (AVL), Automatic Passenger Counting (APC) and Automatic Fare Collection (AFC) systems. These raw data sets are not presented inside the article. These data sets are very rich and each of them includes thousands of records. These data sets can be provided upon the request in Excel format. Route U32 selected in order to conducting this study. This route passes across the most congested sections of the Kuala Lumpur City Centre (KLCC), providing a suitable environment for conducting study on service regularity and reliability. Route U32 is a high-frequency route with high passenger demand. This route has fifty-nine bus stops (almost 30 stops in each direction) and nine buses are operating in this route along an operation day. Table 4 demonstrates route U32 specifications and list of key sops in each direction. All data used in this study collected for time period of September, October and November 2018 (Only weekdays).

Table 1. Route U32 key stops

| **Stop ID** | **Stop Name** | **Order** | **Zone** | **Distance (meter)** |
| --- | --- | --- | --- | --- |
| **West-Bound**  1000970 | HUB TMN DAGANG | 1 | 3 | 0 |
| 1000360 | BLTN KG PANDAN | 21 | 3 | 5347 |
| 1001846 | MAJESTIC/LRT PUDU | 24 | 3 | 7561 |
| 1000958 | HSBC/7 ELEVEN | 28 | 2 | 9375 |
| **East-Bound**  1000958 | HSBC/7 ELEVEN | 28 | 2 | 0 |
| 1001847 | MAJESTIC/LRT PUDU | 37 | 1 | 3894 |
| 1000359 | BLTN KG PANDAN | 41 | 1 | 1590 |
| 1000970 | HUB TMN DAGANG | 60 | 1 | 5706 |

**2. Experimental design, materials and methods**

The simulation model of a single bus route will be used for implementing strategies is based on this framework. The route has a set of locations, each of which can have a set of distributions and a location controller. Distributions are used to model, for instance, segment running times and extra time at the ends of the route. There are two principal types of locations in bus services: terminals and bus stops. In order to run the simulation, route specification, running time distributions, vehicles, demand representation, location controllers, and any additional controller-dependent parameters must be specified. Replication runs follow, each with an initialization and a data collection stage. The simulation model itself is developed in RStudio. RStudio is a free and open-source integrated development environment (IDE) for R, a programming language for statistical computing and graphics. The list of code is attached as appendix A.

Route Specification

Distributions

Vehicles

Demand Representation

Location Controllers

For each replication

Performance Measures

Figure 1. Simulation model activity diagram showing inputs, outputs, and high-level tasks

The former prepares the model to run, while the latter simulates a day of operation and collects performance data. Figure 2 provides a walk-through of the movement of a vehicle in the simulation from the start to the end of a trip.

| 1/6 |  | instantaneous jump  to location 1, first non-terminal location |
| --- | --- | --- |
| 2/6 |  | first segment,  running time from marginal distribution  of segments 1 and 2  (no conditioning) |
| 3/6 |  | second segment,  running time from  conditional distribution of segments 1 and 2, based on segment 1  running time |
| 4/6 |  | third segment,  running time from  conditional distribution  of segments 2 and 3,  based on segment 2  running time |
| 5/6 |  | end of trip, instantaneous jump to  terminal of opposite  direction |
| 6/6 |  | vehicle at terminal... options are taken out of service, begin next trip immediately, or hold and then begin next trip. |

Figure 2. Walk-through of events for a single vehicle


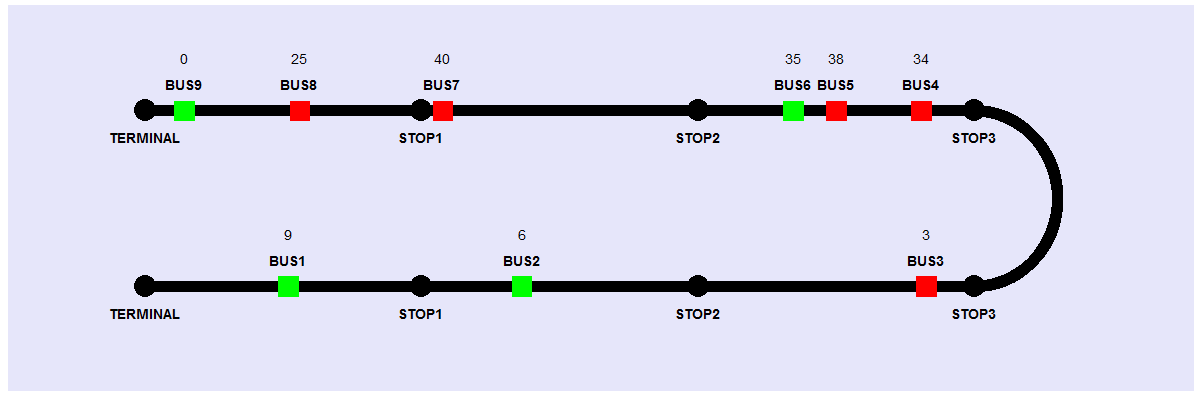


Figure 3. Verification using animated playback

In the context of simulation modelling, verification is the process that ensures that the algorithm works as intended. The simulation model was verified with an interactive debugger, a detailed log file, and animated playback. The interactive debugger allows running the algorithm line by line, verifying the values of variables and the procedural sequence. The detailed log file is a record of actions and decisions executed during runtime. Animated playback shows vehicles moving on the screen throughout the day at an accelerated rate. Figure 3 shows a snapshot of animated playback.

Wilcoxon signed-ranking test was used in order to compare real world situation with simulation models’ out-puts. The Wilcoxon signed-rank test is a non-parametric statistical hypothesis test used when comparing two related samples, matched samples, or repeated measurements on a single sample to assess whether their population mean ranks differ (i.e. it is a paired difference test). It can be used as an alternative to the paired Student's t-test, t-test for matched pairs, or the t-test for dependent samples when the population cannot be assumed to be normally distributed (Randles 1988; Rey & Neuhäuser 2011; Woolson 2008).

Table 2. Validation summary (Wilcoxon sign-ranked test results)

| **Location** | **Dwell time**  **reject (%)** | **Headway**  **reject (%)** | **Running time reject (%)** |
| --- | --- | --- | --- |
| Key stop 1 | 0.3 | 14.7 | ̶ |
| Key stop 2 | 0.9 | 7.7 | ̶ |
| Key stop 3 | 0.2 | 7.8 | ̶ |
| Segment 1 | ̶ | ̶ | 2.8 |
| Segment 2 | ̶ | ̶ | 2.8 |
| Segment 3 | ̶ | ̶ | 4.1 |

The Headway Reliability index based on Stops (HRIS) is designed to capture the operational characteristics at the stop level. Equation below can be used to calculate HRIS. The HRIS is defined such that if its value is zero regularity is perfect, while a value of 1.0 implies bunched arrivals.

$HRIS=\frac{\sum_{i} \left|\frac{H_{i,j}-H_{i,j}^{'}}{H_{i,j}}\right|}{n}$ (eq.1)

Where:

HRIS: headway regularity index based on stops

H_i,j_ = Scheduled headway for bus i at stop j

H^’^_i,j_ = Actual headway for bus i at stop j

n = number of buses serve stop j

Bus bunching and big gap were indicated as causes and consequences of bus service unreliability. Both factors were introduced as cause and also consequence of unreliability. Headways less than 60 seconds and more than twice of related scheduled headway would be considered as bunching and big gap respectively.

The expected wait time on low-frequency routes is a function of on-time performance (schedule deviations) and the proportion of passengers that time their arrival to schedules and passengers that arrive at random [4], [5]. Expected waiting time is calculated using equation below.

$E(w)=\frac{E(h)}{2}\left[1+Cov^{2}(h)\right]$ (eq.2)

where 𝐸[ℎ] is the average headway, and 𝑐𝑜𝑣(ℎ) is the coefficient of variation of headways.

Another measure of passenger wait time for high frequency bus routes is the *excess passenger wait time* (EWT), which measures the difference between actual expected wait time and the expected wait time if headways were as scheduled. It is an appropriate measure to reflect the change in average expected wait time due to poor headway adherence and unreliable service. An average Excess Wait Time is then calculated for each key stop and different headway pattern. Equation 3 gives the general formula for the calculation of EWT:

EWT = AWT ‒ SWT (eq.3)

where the Average Waiting Time (AWT) and the Scheduled Waiting Time (SWT) both are calculated using the equation 2 for average waiting time 𝐸[𝑤].

$EWT=\frac{1}{2}E\left[h_{actual}\right]\left(1+\mathrm{cov}^{2}\left(h_{actual}\right)\right)-\frac{1}{2}E\left[h_{schedule}\right]\left(1+\mathrm{cov}^{2}\left(h_{schedule}\right)\right)$ (eq.4)

Two different types of strategy are implemented in terminal: Scheduled-based departure and headway-based departure. In scheduled-based departure, buses are forced to depart from terminal very on-time. This strategy would be possible through an effective supervision at terminal. Four different types of strategy and their component were implemented on Route U32 and results were recorded by simulation model. Table 3 illustrates the description of adapted strategies. The strategies mechanism are described in Appendix B.

Table 3. Description of combination of strategies

| **Strategies** | **Description** | **Location** |
| --- | --- | --- |
| Strategy 1 | Schedule-based departure from terminal | Terminal |
| Strategy 2 | Headway-based departure from terminal | Terminal |
| Strategy 3 | Previous holding strategy | Key stops |
| Strategy 4 | Prefol holding strategy | Key stops |

Table 4. Effect of terminal dispatch strategies on waiting time

| **Strategy** | **W.T**  **(sec)** | **E.W.T (sec)** | **% of Sch. W.T (300 s)** | **W.T**  **change (%)** | **E.W.T change (%)** |
| --- | --- | --- | --- | --- | --- |
| Headway-based dispatch | 417 | 117 | 139% | -51.22 | -78.91 |
| Schedule-based dispatch | 570 | 270 | 190% | -33.18 | -51.17 |

Table 5. Effect of terminal dispatch strategies on headway regularity index

| **Strategy** | **HRIS 1**  **(% change)** | | **HRIS 2**  **(% change)** | | **HRIS 3**  **(% change)** | **HRIS 4**  **(% change)** | **HRIS 5**  **(% change)** |
| --- | --- | --- | --- | --- | --- | --- | --- |
| Headway-based dispatch | 0.20  (-74%) | 0.32  (-72%) | | 0.52  (-53%) | | 0.62  (-54%) | 0.64  (-58%) |
| Schedule-based dispatch | 0.71  (-7.8%) | 1.144  (-1.3) | | 0.95  (-16%) | | 1.347  (-1.5%) | 1.643  (-0.64%) |

Table 6. Effect of terminal dispatch strategies on big gap and bunching

| **Strategy** | **Big gap**  **(%)** | **Bunching**  **(%)** | **Big gap**  **change (%)** | **Bunching change (%)** |
| --- | --- | --- | --- | --- |
| Headway-based dispatch | 2.00 | 0.0 | -90 | -100 |
| Schedule-based dispatch | 3.5 | 0.0 | -83.33 | -100 |

Figure 4. Comparison of headway-based and schedule-based dispatching strategies

Table 7. Effect of holding strategies on waiting time

| **Strategy** | **W.T**  **(sec)** | **E.W.T (sec)** | **% of Sch. W.T (300 s)** | **W.T**  **change (%)** | **E.W.T change (%)** |
| --- | --- | --- | --- | --- | --- |
| Previous headway | 475 | 175 | 158% | 44% | 68% |
| Prefol headway | 479 | 179 | 159% | 43% | 67% |

W.T= waiting time; E.W.T = excess waiting time; Sch W.T = scheduled waiting time

Table 8. Effect of holding strategies on big gap and bunching

| **Strategy** | **Big gap**  **(%)** | **Bunching**  **(%)** | **Big gap**  **change (%)** | **Bunching change (%)** |
| --- | --- | --- | --- | --- |
| Previous headway | 1 | 0.0 | -95 | -100 |
| Prefol headway | 1 | 0.0 | -95 | -100 |

Table 9. Effect of holding strategies on headway regularity index

| **Strategy** | **HRIS 1**  **(% change)** | | **HRIS 2**  **(% change)** | | **HRIS 3**  **(% change)** | **HRIS 4**  **(% change)** | **HRIS 5**  **(% change)** |
| --- | --- | --- | --- | --- | --- | --- | --- |
| Previous headway | 0.40  (-48%) | 0.44  (-62%) | | 0.59  (-47%) | | 0.67  (-51%) | 0.65  (-58%) |
| Prefol headway | 0.40  (-48%) | 0.44  (-62%) | | 0.60  (-46%) | | 0.69  (-49%) | 0.68  (-56%) |

Figure 5. Comparison of Previous and Prefol headway holding strategies

Table 10. Effect of combination of strategies on waiting time

| **Strategy** | **W.T**  **(sec)** | **E.W.T**  **(sec)** | **% of Sch. W.T (300 s)** | **W.T**  **change (%)** | **E.W.T change (%)** |
| --- | --- | --- | --- | --- | --- |
| 1 & 3 | 464 | 164 | 154% | -45.73 | -70.45 |
| 1 & 4 | 469 | 169 | 156% | -45.14 | -69.54 |
| 2 & 3 | 403 | 103 | 134% | -52.86 | -81.44 |
| 2 & 4 | 409 | 109 | 136% | -52.16 | -80.36 |

Table 11. Effect of combination of strategies on headway regularity index

| **Strategy** | **HRIS 1**  **(% change)** | | **HRIS 2**  **(% change)** | | **HRIS 3**  **(% change)** | **HRIS 4**  **(% change)** | **HRIS 5**  **(% change)** |
| --- | --- | --- | --- | --- | --- | --- | --- |
| 1 & 3 | 0.31  (-59%) | 0.40  (-65%) | | 0.58  (-48%) | | 0.66  (-51%) | 0.64  (-58%) |
| 1 & 4 | 0.31  (-59%) | 0.40  (-65%) | | 0.58  (-48%%) | | 0.67  (-51%) | 0.68  (-56%) |
| 2 & 3 | 0.20  (-74%) | 0.32  (-72%) | | 0.51  (-54%) | | 0.58  (-57%) | 0.57  (-63%) |
| 2 & 4 | 0.20  (-74%) | 0.32  (-72%) | | 0.51  (-54%) | | 0.60  (-56%) | 0.60  (-61%) |

Table 12. Effect of combination of strategies on big gap and bunching

| **Strategy** | **Big gap**  **(%)** | **Bunching**  **(%)** | **Big gap**  **change (%)** | **Bunching change (%)** |
| --- | --- | --- | --- | --- |
| 1 & 3 | 0.0 | 0.0 | -100 | -100 |
| 1 & 4 | 0.0 | 0.0 | -100 | -100 |
| 2 & 3 | 0.0 | 0.0 | -100 | -100 |
| 2 & 4 | 0.0 | 0.0 | -100 | -100 |

Figure 6. Comparison of combination of strategies

Figure 7. Comparison of impact of different strategies on waiting time

Figure 8. Comparison of impact of different strategies on HRIS

**Acknowledgments**

The authors would like to acknowledge the Sustainable Urban Transport Research Centre (SUTRA) of the Faculty of Engineering and Built Environment of the Universiti Kebangsaan Malaysia (UKM) for providing research facilities and the Ministry of Education (MOE) of Malaysia for providing research funding through Project FRGS/2/2013/TK02/UKM/01/1.

**References**

[1] R. F. Woolson, “Wilcoxon Signed‐Rank Test,” *Wiley Encycl. Clin. trials*, 2008.

[2] D. Rey and M. Neuhäuser, “Wilcoxon-signed-rank test,” in *International encyclopedia of statistical science*, Springer, 2011, pp. 1658–1659.

[3] R. H. Randles, “Wilcoxon signed rank test,” *Encycl. Stat. Sci.*, 1988.

[4] M. A. Turnquist, *A model for investigating the effects of service frequency and reliability on bus passenger waiting times*, no. 663. 1978.

[5] L. A. Bowman and M. A. Turnquist, “Service frequency, schedule reliability and passenger wait times at transit stops,” *Transp. Res. Part A Gen.*, vol. 15, no. 6, pp. 465–471, 1981.
