# Supplementary material for: Using simulation model as a tool for analyzing bus service reliability and implementing improvement strategies
Source: PLoS One. 2020 May 7;15(5):e0232799. doi: 10.1371/journal.pone.0232799 (PMC7205290; doi:10.1371/journal.pone.0232799)
Supplement: Attachment — Submitted filename: REPORT ON PONE-D-20-00591PDF.pdf [file pone.0232799.s003.pdf]

REVIEW REPORT ON PONE-D-20-00591

1. INTRODUCTION:
   Lines 52 -73 does not represent an introduction into the subject matter rather it is a brief on what each section of their paper addresses. Author may choose to give a better introductory section on the subject matter then use between 3 to 5 lines to summarize the structuring of their paper.
2. BODY AND RESULT:
   Citation style on line 78 should consistent. Why the use of author names and reference number?
   On line 82, the year of publication for “TCQSM” should be included so as to show currency of thought.
   Sentence on line 87 and 88 does not communicate a complete thought.
   Generally, the discuss on the subject matter isn’t adequate enough to communicate necessary thought by the author.
   Line 127 - 130: Author did not include the basis and/or for selection of the four reliability indicators in the study.
   Also, lines 133 – 143 refer to previous section of the paper that aren’t explicit.
   Presentation of reviewed literature needs to be reviewed to convey sequential flow of thought
   Line 379 also does not give any basis for selection of the four indicators. Author simply referred to literature that isn’t cited
   Tables on lines 454 – 460 seem questionable, probably errors while entering values, otherwise, there is need to justify and/or explain the negative percentage values.

   GENERAL COMMENTS:
   a) The idea presented by the author is original and seem novel but it is greatly undermined by the author(s)’ inability to communicate and accurately present their thoughts.
   b) Literature used haven’t been properly reviewed to show gap filled by the author’s idea.

OVERALL ASSESSESMENT

Paper is suitable for publication with PLOS ONE journals BUT NOT in its current state.

SIGNED:

DR. AKINREFON A.A
