# Supplementary material for: Using simulation model as a tool for analyzing bus service reliability and implementing improvement strategies
Source: PLoS One. 2020 May 7;15(5):e0232799. doi: 10.1371/journal.pone.0232799 (PMC7205290; doi:10.1371/journal.pone.0232799)
Supplement: Attachment — Submitted filename: Editor - initial review paper SIMULATION BUSES.docx [file pone.0232799.s004.docx]

**Editor's initial comments to the paper:**

**Using Simulation Model as A Tool for Analysing Bus Service Reliability and**

**Implementing Improvement Strategies**

The primary objective of this research is to improve the reliability of high-frequency bus service and simulation tools currently used in the public transportation companies. Therefore, a simulation model of high-frequency bus service was developed in order to study the strategies to alleviate it. This study was designed to cover gaps that have been recognized in the literature. According to the authors best knowledge, currently, there is no bus service simulation package available in order to 1) Analyse and measure the level of bus service reliability considering both passengers, and agencies point of view, 2) Implementing corrective strategies and combinations of strategies on bus routes to find out the effect of different strategies, 3) Capturing and comparing the level of reliability before and after implementing any changes on the route 4) Illustrating the movement of buses on a specific route and number of onboard passengers in animation playback and 5) Analysing "Headway Regularity" in term of regularity index, big gap and bunching and excess waiting time. Since all simulation codes and Rstudio files are published with this paper, this simulation model can be expanded by other researchers for further studies in the future. The subject of this research is up-to-date and fundamentally interesting for scholars and practitioners from the field. Thus, from this point of view, the paper likely might have deserved an opportunity to be considered for a possible publication. More importantly, we are dealing with an open-source R environment, which is gradually becoming a leading software environment not only for the simulation but for all kinds of different analyses, modeling spheres, and many other tasks.

However, in the current form, the paper, in general, does not satisfy all rigor requirements that are demanded from Plos One. Although a red clue persists more or less consistent all over the paper, the latter suffers from several detected deficiencies. The editor has detected the following issues, **which should be corrected** prior to continuing a further publishing process:

1. English sentences should be improved.
2. Maybe it would be convenient to add some section or sub-section named “The conceptual framework”, where all consecutive steps of the research should be more clearly emphasized in the form of some block diagram. The latter means a similar structure as it was illustrated in Figure 2, but with more details. Another alternative is to add some pseudo-code clearly demonstrating all research steps and simulation mechanism details.
3. In general, the flow, description, processing, and (intermediate and final) results of all analyses and methods used, might have been, at least in my opinion, better conducted at some places.
4. Please check again if all figures/tables are referenced in the paper.
5. Some figures (e.g., Figure 4) should be improved in the sense of informative content, meaning that the reader immediately understands the main point without even looking at the corresponding text.

Besides the AE comments, here are the comments from the reviewers:

**Reviewer 1 (major revision):**

The paper deals with the topic of bus service reliability, improvement strategies and simulation model for evaluation of proposed strategies. Authors provided another view on topic, however I disagree with the statement that there is a gap in this field. Other authors and transportation companies just used different measurements to evaluate the service reliability. It would be usefull to compare proposed methodologies and strategies with proposals of other authors. The topic is still up-to-date and the proposed methodology and simulations are interesting, however I have few comments and recommendations for the authors.

- English language in the paper should be corrected.

It contains mistakes and misprints.

- The style of references in the paper should be unified. Authors use various styles.

- Table 1 mentioned on the line 90 does not exist.

Table 1 in the paper contains different information.

- Are the values in the table 2 correct? The length of the line in the westbound direction is twice as long as in the eastbound direction?

- The model from the description in the paper seems to be very simple. It would be usefull to describe the simulation model in more details (describe more the used data, describe the simulation tool used for implementing proposed model, etc.).

- The "cov" in the formula (2) should be described in more details.

- Notation in formula (3) should be described in more details.

- Numerical experiments contain only one bus line. It would be usefull to add a discussion about the influence of other factors on the travelling (more bus lines in the system, interchanges between lines and its influence on waiting times and overall travelling time, etc.)

**Reviewer 2 (rejected):**

This paper developed a simulation model of bus service to improve reliability of high-frequency bus service. Four different types of strategies (schedulebased departure from terminal, headway-based departure from terminal, previous holding strategy, prefol holding strategy) were selected according to Route U32 (Kuala Lumpur) specifications. It showed that waiting time has been reduced significantly and headway regularity has been improved.

My main concerns are:

1) This paper developed a simulation model, but it does not specifically explain how the random processes are simulated, such as customer's arrival process etc, and how the traffic/other conditions influence the random process. Also, could you provide other parameters in the experiment such as customer size etc.?

2) The paper defined the expected waiting time in presence of headway variation and excess waiting time on page 18, but why defining it this way? What's the intuition behind this definition and why it is a good way to evaluate the service reliability?

3) Following concern 2), the term mean(h_actual) and cov(h_{actual}) are correlated, so how do you calculate EWT specifically in simulation?

4) What's the running time for each simulation test?

5) Usually there would be multiple routes going through one stop, so could this framework/R package be extended to a traffic network?

6) Is there a way to verify the small gap between the simulation result and the real-world implementations?

**Reviewer 3 (major revision):**

REVIEW REPORT ON PONE-D-20-00591

1. INTRODUCTION:

Lines 52 -73 does not represent an introduction into the subject matter rather it is a brief on what each section of their paper addresses. Author may choose to give a better introductory section on the subject matter then use between 3 to 5 lines to summarize the structuring of their paper.

2. BODY AND RESULT:

Citation style on line 78 should consistent. Why the use of author names and reference number? On line 82, the year of publication for "TCQSM" should be included so as to show currency of thought.

Sentence on line 87 and 88 does not communicate a complete thought.

Generally, the discuss on the subject matter isn't adequate enough to communicate necessary thought by the author.

Line 127 - 130: Author did not include the basis and/or for selection of the four reliability indicators in the study.

Also, lines 133 - 143 refer to previous section of the paper that aren't explicit.

Presentation of reviewed literature needs to be reviewed to convey sequential flow of thought Line 379 also does not give any basis for selection of the four indicators. Author simply referred to literature that isn't cited Tables on lines 454 - 460 seem questionable, probably errors while entering values, otherwise, there is need to justify and/or explain the negative percentage values.

GENERAL COMMENTS:

a) The idea presented by the author is original and seem novel but it is greatly undermined by the author(s)' inability to communicate and accurately present their thoughts.

b) Literature used haven't been properly reviewed to show gap filled by the author's idea.

OVERALL ASSESSESMENT Paper is suitable for publication with PLOS ONE journals BUT NOT in its current state.

According to all reviews, the paper should be likely rejected. However, the Academic Editor does not necessarily share the same opinion. The reason is that the paper, at least from the perspective of the AE, without any doubt, brings certain novelties and contributions. Thus, the AE recommends the authors the following to increase the likelihood of the possible further consideration of the paper: **I suggest to strictly follow all the comments of the reviewers and the AE**.

Academic Editor DD
